# Anatomical and Functional Outcomes after Endoresection and Adjuvant Ruthenium Brachytherapy for Uveal Melanoma: A Single-Center Experience

**DOI:** 10.3390/life13040902

**Published:** 2023-03-29

**Authors:** Cinzia Mazzini, Giulio Vicini, Laura Di Leo, Daniela Massi, Stanislao Rizzo, Fabrizio Giansanti

**Affiliations:** 1 Unit of Ocular Oncology, Neuromuscular and Sense Organs Department, Careggi University Hospital, 50134 Florence, Italy; cinzia.mazzini@unifi.it (C.M.); laura.dileo92@gmail.com (L.D.L.); fabrizio.giansanti@unifi.it (F.G.); 2 Eye Clinic, Neuromuscular and Sense Organs Department, Careggi University Hospital, 50134 Florence, Italy; 3 Department of Neurosciences, Psychology, Drug Research and Child Health, University of Florence, 50121 Florence, Italy; daniela.massi@unifi.it; 4 Section of Pathological Anatomy, Department of Health Sciences, University of Florence, 50139 Florence, Italy; 5 Ophthalmology Unit, Catholic University of the Sacred Heart, Fondazione Policlinico Universitario A. Gemelli, 00168 Rome, Italy; stanislao.rizzo@gmail.com; 6 Consiglio Nazionale delle Ricerche (CNR), 56124 Pisa, Italy

**Keywords:** uveal melanoma, endoresection, adjuvant radiotherapy

## Abstract

Purpose: To evaluate the anatomical and functional outcomes of endoresection and adjuvant ruthenium (Ru)-106 brachytherapy for uveal melanoma (UM). Methods: Retrospective case series of 15 UM patients (15 eyes) treated at our center (Careggi University Hospital, Florence). Results: Six patients (40%) were male and nine were female (60%). The mean age of patients at the time of treatment was 61.6 years (±18.47). The mean BCVA at baseline was 20/76. In all cases UM originated from the choroid. The mean tumor thickness at baseline was 7.20 mm (±2.01), and the mean largest basal diameter was 11.24 mm (±2.20). A concurrent retinal detachment was diagnosed in 11 patients (73.3%). Two patients (13.3%) showed vitreous seeding at baseline. Eleven patients (73.3%) were treated with primary endoresection, while four patients (26.7%) were treated with a “salvage endoresection” after primary treatment failure (previous radiation treatment). The mean follow-up time was 29.9 months (±10.6). Thirteen out of fifteen patients were alive and showed no evidence of local recurrence or distance metastasis at the last follow-up visit. The treatment achieved local control of the disease in 14 out of 15 cases (93.3%). In one case, the patient underwent enucleation for disease recurrence. The overall survival rate at the end of the follow-up was 93.3%. The mean BCVA at last follow-up visit was 20/70. Treatment was well tolerated, without significant complications. Conclusions: Endoresection and adjuvant Ru-106 brachytherapy is a valuable conservative option for selected UM patients and can be used both as a primary treatment and as a salvage therapy. It can control melanoma and avoid enucleation, reduce radiation-related complications, and provide tumor tissue for chromosomal analysis and prognostic testing.

## 1. Introduction

Although rare, uveal melanoma (UM) is the most common primary intraocular tumor in the adult population, with standardized incidence rates of 1.3–8.6 cases per million per year in Europe, depending on latitude [1,2]. It arises from uveal melanocytes, with a prevalent choroidal location (85–90%) [2]. Historically, enucleation of the affected eye was considered the only appropriate treatment for UM, however, more conservative treatments were developed over the last three decades to improve cosmesis and the possibility of visual preservation [3,4]. Despite developments in eye-sparing therapies, with good local control of the disease, melanoma-related mortality has remained constant: this suggests that successful local treatment does not affect survival rates [4]. Since the publication of the Collaborative Ocular Melanoma Study, radiation treatment has become the gold standard for UM [3,4,5,6]. Radiotherapy can be administered in the form of brachytherapy, used as first choice treatment for smaller tumors, or proton beam radiotherapy and stereotactic radiosurgery, which are preferably used in medium and large tumors [3,6]. The surgical resection of the tumor is another viable option for selected patients with UM [7,8,9,10,11,12,13,14,15,16,17]. In consideration of the multiple treatment options currently available, treatment should be highly individualized depending on several factors related to the tumor itself, such as dimensions, location, involvement of the surrounding structures and activity, and other factors related to the patient including status of the fellow eye, age, general health, and psychological status.

UM with a tumor thickness of up to 6 mm can be successfully managed with ruthenium brachytherapy alone, and high tumor control rates can be achieved. However, juxtapapillary UM and UM with a tumor thickness greater than 6 mm remain challenging in terms of tumor control and the occurrence of vision-threatening complications and globe salvage [18,19]. In such cases, iodine brachytherapy or teletherapy should be considered. Despite the effective local control and eye preservation, stereotactic radiosurgery and proton beam radiotherapy for UM can secondarily be impaired by high percentage of severe vision-threatening side-effects, as radiation retinopathy, optic neuropathy, and secondary glaucoma, resulting from the irradiation of the surrounding healthy tissues [20,21,22,23,24,25,26,27]. Secondary glaucoma significantly affects quality of life and is the second most frequent complication leading to enucleation after local recurrence [28,29,30]. Radiotherapy remains the standard of treatment in UM, however, due to the high morbidity deriving from the techniques adopted to treat the larger tumors, research continues for local therapies that could achieve the goal of tumor control with less collateral damage to normal ocular structures and vision.

The surgical resection of UM was developed with the intent to control the tumor while preserving the globe, conserve as much useful vision as possible, and sparing the patients the effects of radiotherapy [15]. It can be performed through a trans-scleral approach (exoresection) or through an internal approach utilizing pars plana vitrectomy (endoresection). Endoresection surgery has been utilized over the last three decades with promising results, but only the advances in vitreoretinal surgery technology made in recent years have significantly improved the technique [16,17]. In recent years, endoresection of the tumor utilizing pars plana vitrectomy combined with adjuvant ruthenium brachytherapy has proven to be an effective treatment for selected patients with large choroidal melanoma [12,17,31]. It may be performed both as a primary intervention or as a salvage procedure whenever other conventional primary or salvage procedures are not suitable.

We retrospectively evaluated the treatment outcomes following endoresection and adjuvant ruthenium (Ru)-106 brachytherapy for UM in our center. The aim of this paper is to describe our experience in the treatment of large UM using a combined approach of surgical resection with brachytherapy. We included both patients who underwent endoresection as primary treatment, and patients who underwent this procedure as a salvage treatment after a radiotherapy failure.

## 2. Materials and Methods

We conducted a single-center observational retrospective study on patients with UM who underwent endoresection and adjuvant Ru106-brachytherapy between December 2018 and July 2021. The study was conducted at the Unit of Ocular Oncology, Neuromuscular and Sense Organs Department, Careggi University Hospital, Florence, Italy, and was performed according to the current version of the Declaration of Helsinki (52nd WMA General Assembly, Edinburgh, Scotland, UK, October 2000). Informed consent was obtained from all patients prior to therapy: all patients were actively involved in treatment decisions and possible complications were widely discussed. The study was approved by the Careggi University Hospital Research Ethics Board. We included both patients with newly diagnosed UM who underwent endoresection as primary treatment, and UM patients who underwent this procedure as a salvage treatment after primary treatment failure. In the latter case, the primary treatment was radiotherapy, as Ru-106 brachytherapy or stereotactic radiotherapy.

Data were collected regarding patient demographic data, tumor features, and treatment characteristics. Each patient was evaluated by a multidisciplinary panel consisting of an ophthalmologist, radiation oncologist, oncologist.

At the baseline each patient underwent best-corrected visual acuity (BCVA) measurement with Snellen charts, intraocular pressure (IOP) measurement with Goldmann applanation tonometer, dilated fundus examination, ultra-widefield fundus photography, green light fundus autofluorescence, standardized A- and B-scan ocular ultrasonography and swept-source optical coherence tomography (SS-OCT).

UM patients selected for endoresection via pars plana vitrectomy combined with adjuvant Ru-106 brachytherapy were patients with large lesions (tumor thickness > 6 mm or largest basal diameter > 16 mm), not eligible for more conservative treatment (Ru106 brachytherapy alone) due to the tumor dimensions. Patients for whom gamma knife treatment and proton beam radiotherapy were excluded (e.g., due to the difficulty of fitting the Leksell frame on patients with large anteroposterior diameter of the skull, due to patient preferences, or due to logistic problems), as were patients with particular clinical conditions (e.g., only one functioning eye) where the risk–benefit balance was in favor of an approach that allowed the conservation of as much useful vision as possible.

We excluded patients with signs of scleral invasion or distant metastases.

After ophthalmologist evaluation, the opportunity to perform this type of treatment was discussed in a multidisciplinary tumor board (including ophthalmologists, radiation oncologists, and oncologists). Tumor characteristics at baseline were recorded including tumor dimensions (thickness and largest basal diameters), tumor shape (e.g., dome-shaped, mushroom-shaped, or plateau-shaped mass), location with respect to the equator (posterior/anterior) and presence of subretinal fluid/retinal detachment.

During each monitoring visit after treatment, patients underwent a complete ophthalmological examination, fundus photography, and A- and B-scan ultrasound.

Follow-up data were collected regarding local tumor control, treatment complications on anterior and posterior segment, need of enucleation, occurrence of metastases, and survival status.

### Surgical Procedure

All the surgical procedures were performed under general anesthesia by experienced vitreoretinal surgeons. Anesthesia was induced with propofol. The surgeries were made using the Ngenuity 3D Visualization System (Alcon Laboratories, Fort Worth, TX, USA) mounted on the OPMI Lumera 700 microscope (Carl Zeiss Meditec AG, Jena, Germany). A standard three-port, 25-gauge pars plana vitrectomy with valved trocars (Constellation, Alcon Laboratories, Fort Worth, TX, USA) and lens extraction with IOL implant was performed in all cases. Chandelier endoilluminator was inserted to facilitate bimanual maneuvers. Intraocular pressure was increased to 80 mmHg to induce temporal closure of the choroidal circulation, to prevent bleeding and hematogenous dissemination. Removal of the melanoma with the vitrectomy probe was begun at the tumor apex until the scleral bed was free of tumor. Diode endolaser was performed on the scleral bed to destroy residual tumor cells.

Two different endoresection approaches were used to perform melanoma endoresection: a “retinal flap” approach (folding the retina away) or a “transretinal resection” approach (cutting the tumor and the overlaying retina).

**Figure 1:**
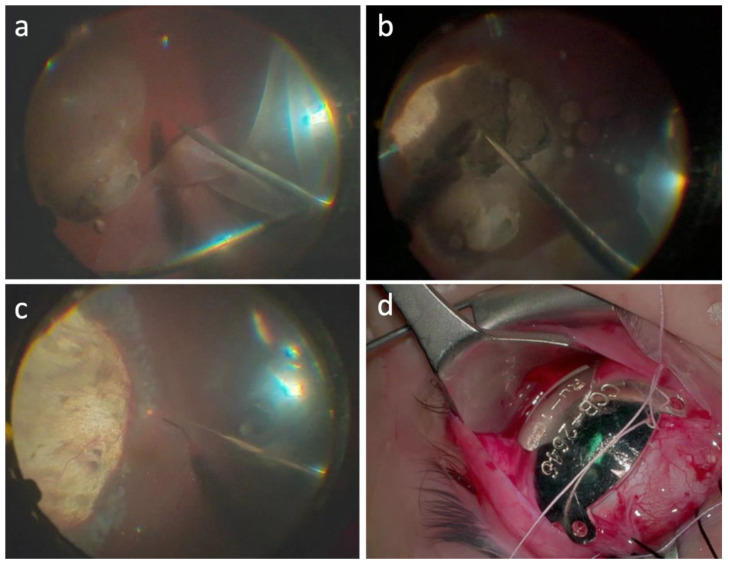
Endoresection surgical procedure in case #1. (**a**) A 23-gauge pars plana victrectomy was performed, then, access to the lesion was gained making a peripheral retinectomy of 180°, from 12 to 6 o’clock, and folding the retina away from the tumor. (**b**) Next, confluent endolaser photocoagulation was performed 360° around the lesion, followed by resection of the tumor with the 23-gauge probe. The intraocular pressure was increased to 80 mmHg for 5 min to induce temporal closure of the choroidal circulation, to prevent bleeding and hematogenous dissemination. Removal of the melanoma with the vitrectomy probe was begun at the tumoral apex until the scleral bed was free of tumor. (**c**) Continuous endolaser was performed on the scleral bed to destroy possible malignant residual cells. Then, the retina was reattached with perfluorocarbon liquid and endolaser photocoagulation was performed around the tumor margins. A fluid–air exchange was made, and the eye was filled with silicone oil 1300 cs. Closure of the sclerotomies was performed using cryotherapy. (**d**) At the end of the surgery, Ru-106 plaque was sutured onto the sclera, covering the whole resection area, with the aim of destroying residual tumor cells.

In the “retinal flap” approach a 39-gauge needle was mounted on a 10 cc syringe, containing only balancing saline solution (BSS) connected with the viscous fluids injecting circuit of the vitrectomy machine. The fluid was injected under the retina to induce a retinal detachment in the interested area. A wide retinotomy (120–180°) was performed in the retinal periphery using the vitrectomy probe. The retinal flap obtained was flipped to expose the choroidal melanoma. Endophotocoagulation was then performed at the edge of the lesion and careful endodiathermy was performed to close the tumor feeder vessels and decrease the risk of hematogenous dissemination and bleeding (Figure 1a). The tumor was removed using the vitrectomy probe and endophotocoagulation was applied to the surgical coloboma bed (Figure 1b). The retina was reattached over the choroidal coloboma using perfluorocarbon liquid, and a laser retinopexy was performed at the edge of the retinotomy area (Figure 1c). In cases where UM was infiltrating the retina, a “transretinal resection” approach was employed, where both the retina and the neoplasm were simultaneously removed with the vitrectomy tube to avoid residual cells in the vitreous chamber. Retinopexy was performed by applying endolaser photocoagulation to the retinotomy edges.

A perfluorocarbon–silicon oil direct exchange was carried out at the end of the surgery. Fluid–air exchange has never been used to avoid venous air embolization. In all cases, a standard silicon oil 1300 cs was used as endotamponade. In all cases the retina remained fully attached at the conclusion of surgery. At the end of the surgery, a Ru-106 plaque was sutured onto the sclera, covering the whole resection area, with the aim of destroying any residual tumor cells (Figure 1d). The Ru-106 plaque was removed the next day after delivering 80 Gy to a depth of 3 mm. In all cases, the tissue material collected during the surgery was submitted for pathological and cytogenetic examination. All patients were instructed to maintain a facedown position for 1 week. Silicone oil removal was performed 3 months later. Follow-up examination was performed at 1 week, 1 month, 3 months, and every 6 months thereafter. Ophthalmoscopic examination and screening for metastasis were carried out every 6 months.

## 3. Results

### 3.1. Demographic and Clinical Characteristics

The study included 15 eyes of 15 patients affected by UM and treated with endoresection and Ru-106 brachytherapy between December 2018 and July 2021. Six patients (40%) were male and nine were female (60%). The mean age of patients at the time of treatment was 61.6 years (±18.47; ranging from 22 to 92). The mean BCVA at baseline, before endoresection surgery, was 20/76 (ranging from hand motion to 20/20). Mean intraocular pressure prior the treatment was 14 mmHg.

In all cases, UM originated from the choroid. The mean tumor thickness at baseline was 7.20 mm (±2.01), with a minimum of 2.63 mm and a maximum of 11.7 mm, and the mean largest basal diameter was 11.24 mm (±2.20), with a minimum of 7.5 mm and maximum of 15.4 mm. A concurrent retinal detachment was diagnosed in 11 patients (73.3%). Two patients (13.3%) showed vitreous seeding at baseline (case #3 and case #14). The tumor was located posterior to the equator in all cases, with 60% of cases in the temporal sectors. All the patients were phakic at the time of surgery and six (40%) had cataracts of different degrees. No patients had clinical or radiological evidence of metastasis at the time of treatment. Characteristics of the patients included in the study are summarized in Table 1.

### 3.2. Treatment Outcomes

**Figure 2:**
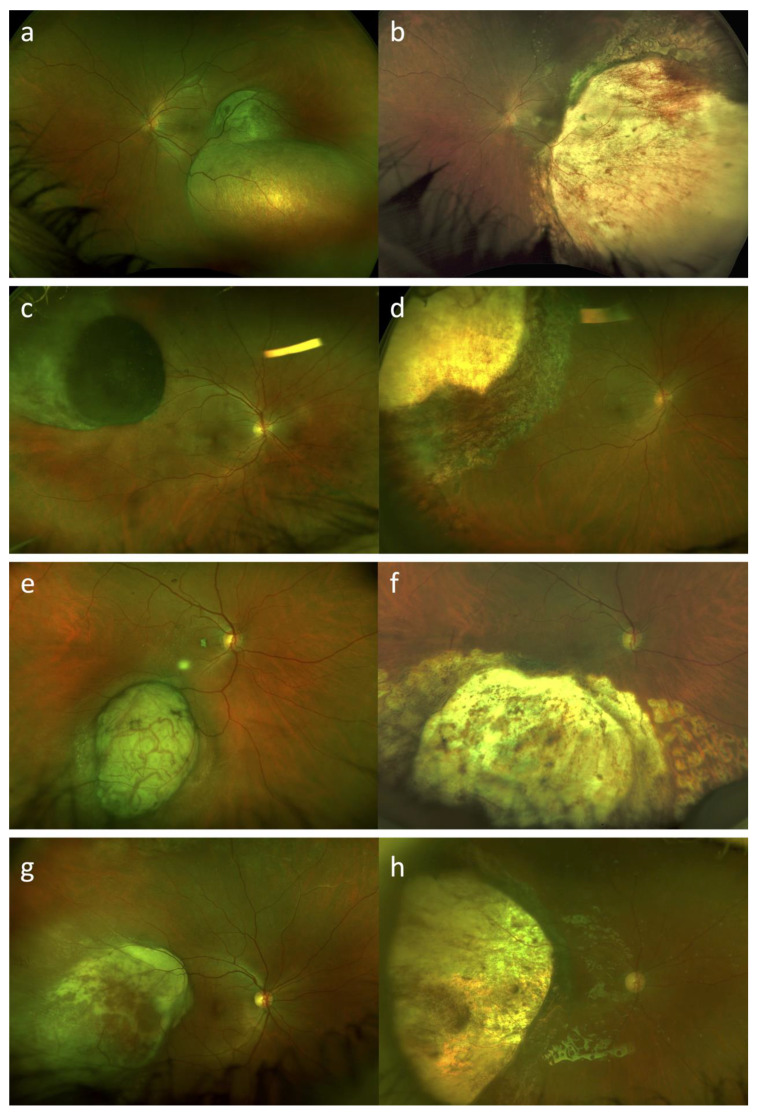
Ultra-widefield fundus photography (Daytona, Optos, Dunfermline, UK) of uveal melanomas before and after treatment with endoresection and adjuvant Ru-106 brachytherapy. Case #1, case #3, case #4, and case #15 at baseline (**a**,**c**,**e**,**g**) and after treatment (**b**,**d**,**f**,**h**), respectively. In postoperative photos we can clearly see the surgical choroidal colobomas.

Eleven out of fifteen patients (73.3%) were treated with primary endoresection and adjuvant Ru-106 brachytherapy. Four out of fifteen patients (26.7%) were treated with a “salvage endoresection” after primary treatment failure: in two cases stereotactic radiotherapy (gamma knife) was performed as primary treatment, and in the other two cases Ru-106 brachytherapy was performed as primary treatment. Thirteen out of fifteen patients (86.7%) received cataract surgery and IOL implantation during endoresection procedure, in one case, who was the youngest patient included in this series (case #1), cataract surgery was performed during silicone oil removal due to lens opacification. In one case (case #15) during silicone oil removal surgery, an inferior retinal detachment occurred, and silicone oil was used as endotamponade again; silicone oil removal was performed after a further 3 months with success. Figure 2 shows ultra-widefield fundus photography of four cases of uveal melanomas included in our series, before and after treatment.

In six cases (40%) endoresection was performed with a “retinal flap” approach, while in nine cases (60%) a “transretinal resection” approach was performed.

The mean follow-up time was 29.9 months (±10.6), with a minimum of 12.7 months and a maximum of 45.2 months. Thirteen out of fifteen patients were alive and showed no evidence of local recurrence or distance metastasis at the last follow-up visit. The treatment achieved local control of the disease in 14 out of 15 cases (93.3%). In one case, in which endoresection was performed as a “salvage therapy” to treat a primary treatment failure after Ru-106 brachytherapy, the patient underwent enucleation for disease recurrence at the surgical coloboma margins 25 months after endoresection surgery. The overall survival rate at the end of the follow-up was 93.3%.

In one case, in which endoresection was performed one year after the brachytherapy treatment, the patient developed liver metastases (6 months after treatment) and died for metastatic disease.

**Figure 3:**
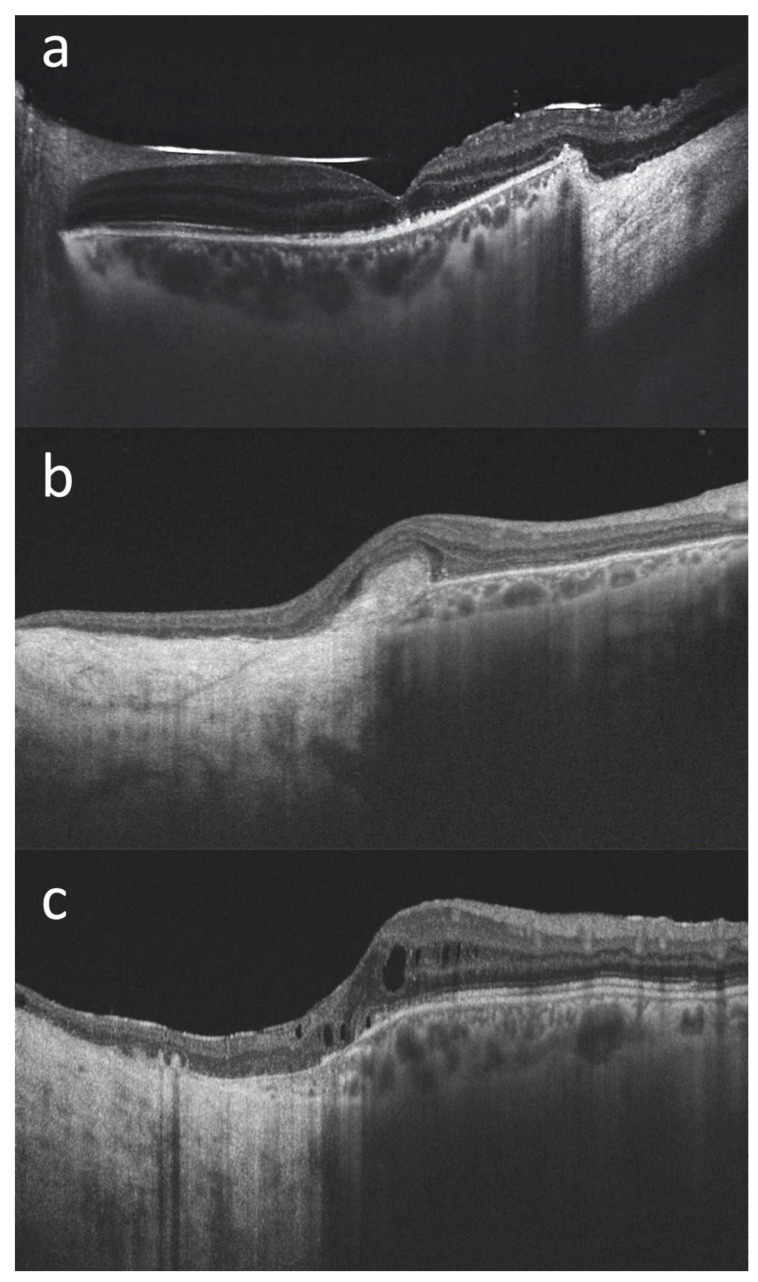
Macular optical coherence tomography (DRI OCT Triton; Topcon Corporation, Tokyo, Japan) scans of three patients with choroidal melanoma involving the posterior pole who have undergone endoresection with preservation of the retina overlying the lesion ((**a**): case#1, (**b**): case #2, (**c**): case #15). We can see the transition from the normal retina and choroid to the surgical choroidal coloboma, with the retina directly overlying the hyperreflective sclera, in all cases. The foveal retinal profile is relatively well preserved in (**a**), while it is altered in (**b**,**c**) where the surgical coloboma reaches the macula. In case (**b**) we can see an hyperreflective subfoveal fibrous scar. In case (**c**), a mild cystoid macular edema is noticeable. The silicone oil meniscus and irregularities of the subfoveal outer retinal layers are also visible in (**a**) (the scan was acquired prior to the silicon oil removal). In all three cases a useful visual acuity was maintained at the last follow-up visit (20/100 in case #1, 20/125 in case #2, and 20/32 in case #15).

The mean BCVA at last follow-up visit was 20/70 (ranging from counting fingers to 20/20). Mean IOP at last follow-up was 16 mmHg, with three out of fifteen patients on IOP-lowering medication (case #6, case #8 and case #14). When the lesions extended posteriorly, surgical resection involved the macular region. In these cases (for example case #1 and case #15—Figure 3), it was still possible to maintain a useful visual acuity.

### 3.3. Histophatological and Cytogenetic Analysis

UM was verified in all cases with histopathological analysis conducted on the tissue material collected during the surgery. Cytogenetic analysis with fluorescence in situ hybridization (FISH) was carried out in all patients treated with endoresection and brachytherapy. Nine out of fifteen (60%) patients showed evidence of genetic imbalance with monosomy 3, included the patient who was enucleated for local recurrence. It is noteworthy that, regarding the patient who underwent enucleation due to local recurrence, the first cytogenetic exam performed at the time of primary treatment (Ru-106 brachytherapy) did not show sign of monosomy 3 and the second examination performed on the material obtained with endoresection was also negative, while monosomy 3 was found at the third and last examination performed after enucleation. Regarding the chromosome 8 status, 8q gain was found in 6 out of 15 (40%) patients. Chromosome 6 was evaluated in six patients: two of them showed no alterations, one case showed 6q loss, three cases showed 6p gain and two cases showed both alterations. Cytogenetic analysis results are summarized in Table 1

### 3.4. Treatment Complications

Overall, the treatment was well tolerated in our series. In only one case a retinal detachment occurred after the silicon oil removal (case #15). Other complications included three cases of postoperative ocular hypertension with need of antihypertensive medication (case #6, case #8 and case #14), and one case of radiation retinopathy (case #13). No major surgical complications were observed in our series. None of our patients ended up with phthisis bulbi or needed enucleation for major surgical complications. There were also no systemic complications.

## 4. Discussion

We described our experience in the treatment of large UM with endoresection and adjuvant Ru-106 brachytherapy, both as primary or as salvage treatment after prior radiation therapy. The results of our series suggest that the use of this approach in selected UM patients may achieve good anatomical and functional outcomes, without significant treatment-related complications.

The internal resection of UM was performed for the first time in 1986 by Peyman and Cohen to treat tumors located in close proximity to the optic disc [32]. Subsequently, Lee et al. (1993) and Damato et al. (1998) reported their results in treating patients using the same procedure [7,33]. Initially this surgical technique was limited to posterior tumors for whom radiotherapy would be technically difficult, and was then extended to large posterior tumors for whom radiation would cause severe vision loss and enucleation would typically be recommended for [9,11,12,16]. In the literature, endoresection was more often described as a primary treatment in radiotherapy-naive patients, however, it was also used to treat patients with local recurrence following radiotherapy, when a large amount of necrotic tissue was expected (as is the case following gamma knife irradiation or proton beam irradiation of large tumors), in case of persistent large exudative retinal detachment following radiation, or in case of intractable vitreous hemorrhage and ghost cell glaucoma [7,8,12,17,34,35,36,37,38].

The results of our study are consistent with those of other experiences reported in the literature. The mean follow-up time in our series was 29.9 months. Thirteen out of fifteen patients (86.7%) were still alive and showed no evidence of local recurrence or distance metastasis at the last follow-up visit. In our study, no local recurrences were found after primary endoresection while a single case of recurrence was found after a salvage procedure. In this case, secondary enucleation was performed.

Reported rates of long-term local recurrence and enucleation after endoresection for UM are quite variable, ranging from 0% to as high as 23% [17]. Damato et al. reported no cases of recurrent disease in 41 primary endoresection surgeries, and one case after salvage endoresection [7]. Kertes et al. reported one local recurrence in 32 patients (3.1 %) with a mean follow-up time of 3.5 years [39]. Karkhaneh et al. detected one recurrence at the margins of the surgical coloboma and another new tumor focus in a study which included 20 patients (10%) with a mean follow-up time of 89.5 months [9]. Garcia Arumi et al. found local recurrence rates of 5.1% and 6% at 3 years and 5 years, respectively [12]. Susskind et al. reported 5- and 10-year actuarial rates of recurrence of 22.7% and 29.2%, respectively [31]. Caminal et al. found a 5-year recurrence rate of 12.2% [10]. Rice et al. found a recurrence rates of 18.2 % [40].

In our series, only one patient developed liver metastases and died as a consequence. The overall survival rate at the end of the follow-up was 93.3%. The patient who developed metastatic disease received endoresection as a salvage treatment after local recurrence occurred one year after Ru106-brachytherapy.

Despite the initial concerns about the risks of local or metastatic spread, the available data show that endoresection achieves survival outcomes comparable to those obtained with brachytherapy [17]. However, the technical complexity of this surgical procedure limits its use to a small number of specialized centers, especially in cases in which primary radiotherapy is deemed unsuitable.

The literature shows cancer-specific mortality rates ranging from 0% to 20%, albeit with variable follow-up outcomes [7,9,10,11,12,13,14,31,39,40,41,42]. Actuarial (Kaplan–Meier) survival rates at 5 years range from 90.9% to 100%, while at 10 years, survival rates range from 57.9% [28] to 97.6% [12,31]. These data are similar to those from the COMS study, in which 5-year overall and disease-specific mortality rates for medium-sized melanomas were 20% and 10%, respectively [43].

Only two retrospective studies have compared primary endoresection to brachytherapy for UM. Both studies found no significant differences in overall survival or disease-specific survival [10,40]. Rice et al. compared the outcomes of Iodine-125 brachytherapy (n = 148) and endoresection (n = 22) in the conservative treatment of medium sized choroidal melanoma (2.5–10 mm in height and up to 16 mm in the widest diameter) [40]. This study suggests that endoresection of selected tumors may achieve better visual outcomes than brachytherapy. Visual acuity of 6/18 or better was maintained in 41% of the endoresection group and 35% of the brachytherapy group. The likelihood of achieving a final visual acuity of better than 2/60 was 22% higher in the endoresection group (*p* = 0.034). Local recurrence rate was higher in the endoresection group than the brachytherapy group (18.2% vs. 14.9%), but was not statistically significant.

Caminal et al. compared primary endoresection to primary I-125 brachytherapy in a study in which cases were matched according to tumor height and postequatorial tumor location. No statistically significant differences between the two groups were observed in overall survival, metastasis-free survival, visual acuity, or eye retention [10].

UM surgical resection can be performed solely or in combination with preoperative or adjuvant radiotherapy. The use of an adjunctive ruthenium plaque has been initially suggested as a tool to improve local control, considering the possibility that tumor cells remain in the scleral bed or along the resection edges leading to tumor recurrence [7,44,45], and it is now generally accepted that adjuvant radiation after tumor resection can reduce the incidence of both recurrences and metastases [18].

A recent study conducted by Relimpio-López et al. evaluated the clinical outcomes of a combined approach (resection surgery and brachytherapy, conducted simultaneously or deferred) in management of anterior-located UM, affecting mainly the iris and ciliary body [45]. This experience included 26 patients treated with different combined techniques—exoresection followed by brachytherapy or endoscopy endoresection for ciliary body tumors—and showed them to be an effective and safe approach in selected patients, as long as strict follow-up is conducted after surgery.

The main concern about endoresection technique is that surgical manipulation of the tumor with the vitreous cutter might theoretically result in systemic and local dissemination of tumor cells. However, as more data become available in the literature, this risk appears to be lower than originally believed and endoresection proves to be a safe procedure in terms of tumor control.

Circulating tumor cells in UM are more frequently detected in the metastatic stage compared to the early stage [46]. In the early stage, most patients in whom circulating tumor cells were detected had adverse prognostic risk factors (Class 2 status by gene expression profiling and monosomy 3 with increased risk of distant metastasis and worse clinical outcomes). The high detection rate of circulating tumor cells in UM patients suggests that the metastatic spread precedes initial diagnosis and treatment [47,48,49]. The rate of circulating tumor cells in UM does not appear to be influenced by the treatment modality. Suesskind et al. conducted a prospective study on 81 patients with UM demonstrating that no significant differences could be found in circulating melanoma cells among different treatment techniques, before and after treatment [50]. This study included 19 patients who underwent endoresection. None of the single therapeutic modalities included in the study (enucleation, endoresection, stereotactic radiotherapy, brachytherapy and transpupillary thermotherapy) was associated with changes in circulating melanoma cells values in the systemic circulation (all *p* > 0.20).

Overall, in our series the visual acuity was preserved and slightly increased at the last follow-up visit, with a mean BCVA of 20/70, while the preoperative BCVA was 20/76. For functional preservation purposes, in addition to the size of the tumor, a crucial aspect is its location. Regarding the dimensions, the mean tumor thickness was 7.20 mm, and the mean largest basal diameter was 11.24 mm, with most lesions in the “large” class of the COMS classification. On the other hand, regarding location, all the tumors included in our series were posterior to the equator, and in 60% of cases, the tumor was located in temporal sectors. In cases of endoresection for temporally located tumors, the macula and visual function are more likely to be affected [12]. In our case series, however, we observed an overall slight increase in mean BCVA, despite the localization of the tumors and the treatment received, which included adjuvant radiotherapy. In this regard, it should be noted that most of the patients had an exudative retinal detachment which was fixed with treatment, furthermore the increase in visual acuity may be also justified by the cataract extraction which was routinely performed in each case (all patients were phakic and six of them had cataracts). 

Compared to others conservative approaches, endoresection surgery has the undoubted advantage to providing tumor tissue for chromosomal analysis and prognostic testing in all cases. In recent years, several studies have focused on the identification of prognostic factors for UM, highlighting the importance of cytogenetic and molecular genetic characteristics of the disease [51,52,53,54]. One of the most important chromosomal abnormalities associated with an increased risk of metastasis is the loss of a copy of chromosome 3 (monosomy 3), with a reduction of 5-year survival from approximately 100% to 50% [51,52]. Other abnormalities, such as 8q gain and 1p loss correlate, with poorer survival [51,53]. In our series, cytogenetic analysis was performed in all cases, and monosomy 3 was detected in 9 out of 15 (60%) patients. These data suggest a biologically aggressive disease in most cases, in a higher percentage than other experiences reported in the literature. Despite this, no higher recurrence and metastasis rates than other studies were observed.

The main complications of endoresection surgery include intraoperative bleeding, postoperative retinal detachment, commonly related to proliferative vitreoretinopathy, local tumor recurrence, and rarely fatal air embolism following fluid–air exchange [11,55]. In our series the treatment was very well tolerated without significant complications.

Previously, air was used to flatten the retina in this surgical procedure; however, this method was abandoned after the report of intraoperative and early postoperative death caused by air embolism [55,56]. It is known that air embolism can develop after endoresection for choroidal melanoma, despite avoiding air infusion [57,58]. Ruschen et al. hypothesized that a gas embolism was due to the entry of perfluorooctane into the bloodstream with the formation of gas bubbles in the pulmonary circulation, and the association with an increased vapor pressure of perfluorooctane [57]. In our case we always used perfluorodecalin, which has been reported to enter circulation during endoresection without systemic complications [58]. No significant complications associated with radiotherapy were observed in our series. It must be considered that brachytherapy was conducted with the lowest dose, sufficient to eliminate residual cells on the scleral bed, and with the vitreous chamber filled by silicon oil which appears to reduce the radiation-induced collateral damage on the healthy eye structures [59].

This study addresses some considerations that need to be taken into account. We are aware that this is a small retrospective series of 15 carefully selected UM patients, however, the results have been promising both in terms of disease control and functional preservation, confirming safety and efficacy data reported in the literature. The average follow-up time in our series was 29.9 months and the long-term risks of local recurrence and systemic spread remain uncertain, however, even the endoresection experiences in the literature in which the follow-up times were very long have not shown differences in terms of disease control for this procedure compared to other methods [12,17]. In any case, it will be interesting to observe the results in terms of disease control at a more extended follow-up in our series.

Our case studies also included two young patients (22 and 31 years at the time of treatment, case #1 and case #12) with a long life expectancy who underwent primary endoresection. These two patients presented large, posteriorly located lesions for which radiation treatment could have led to significant complications, and a non-negligible risk of secondary enucleation. We believe that the anatomical preservation of the eye in this case takes on particular importance, considering also the greater psychological distress of younger patients following eye removal [60]. These two young patients not only retained the eye, but also useful vision (BCVA 20/100 and 20/80 at the last follow-up visit), unchanged from baseline. This result appears to be extremely favorable, considering the size of the lesions and the localization, and we believe that it would have been difficult to obtain with other available treatment modalities.

## 5. Conclusions

In conclusion, we described the successful management of large choroidal melanomas using transvitreal endoresection and adjuvant brachytherapy. This highly complex surgical procedure is a valuable conservative option for selected patients in whom other forms of conservative treatment are likely to cause severe ocular complications and can be used both as a primary treatment and as a salvage therapy. It allows us to control melanoma and avoid enucleation, reduces radiation-related complications, and provides tumor tissue for chromosomal analysis and prognostic testing. Further studies with more patients and longer follow-up are needed to establish the safety of this procedure and the risks of recurrence, metastasis, and enucleation.

## Figures and Tables

**Table 1:** Patient, tumor and treatment characteristics.

ID	Age *	Sex	Prior Treatments	BD (mm) **	TK (mm) **	Tumor Quadrant	RD **	Surgical Approach	BCVA **	BCVA ***	Eye Retention ***	M3	8qG	MT ***	Follow-Up Time (Months)
#1	22	M	None	13.0 × 15.0	6.9	IT	Y	RF	20/100	20/100	Y	Y	N	N	45.2
#2	56	M	None	10.2 × 11.3	7.4	ST	Y	RF	20/100	20/125	Y	Y	Y	N	42.4
#3	61	M	None	5.5 × 7.5	8.0	T	N	TR	20/20	20/20	Y	Y	N	N	40.3
#4	68	F	None	10.7 × 11.6	6.5	IT	N	RF	20/50	20/63	Y	N	N	N	39.8
#5	72	M	BT	10.9 × 15.4	6.4	T	Y	RF	20/100	/	N	Y	Y	N	38.9
#6	73	M	None	8.8 × 9.7	6.2	INa	Y	TR	HM	20/400	Y	N	N	N	36.8
#7	72	F	None	10.77 × 7.5	6.6	IT	Y	TR	20/63	20/63	Y	N	N	N	20.2
#8	82	M	None	11.5 × 11.0	7.0	Na	Y	TR	20/100	20/400	Y	Y	N	N	16.0
#9	92	F	None	13.4 × 12.3	10	T	Y	TR	20/400	20/100	Y	Y	Y	N	21.9
#10	63	F	None	11.6 × 11.3	11.7	I	Y	TR	20/800	CD	Y	Y	N	N	12.7
#11	70	F	BT	11.0 × 8.5	5.6	Na	Y	TR	20/80	20/50	Y	N	Y	Y	28.2
#12	31	F	None	12.4 × 10.9	7.6	T	Y	RF	20/80	20/80	Y	N	Y	N	19.5
#13	67	F	GK	8.9 × 8.5	8.0	Na	Y	TR	20/400	CD	Y	N	N	N	31.0
#14	45	F	None	8.7 × 7.1	7.1	SNa	N	TR	20/50	20/50	Y	Y	N	N	21.6
#15	50	F	GK	9.9 × 8.3	2.63	T	N	RF	20/50	20/32	Y	Y	Y	N	34.5

* age in years at time of treatment; ** at baseline; *** at last follow-up; BD: basal diameters; TK: thickness; RD: retinal detachment; BCVA: best-corrected visual acuity; M3: Monosomy of chromosome 3; 8qG: Gain of chromosome 8q; MT: Metastatic tumor; BT: Ruthenium-106 brachytherapy; GK: gamma knife; IT: inferotemporal; ST: superotemporal; T: temporal; INa: inferonasal; Na: nasal; I: inferior; SNa: superonasal; RF: retinal flap approach; TR: transretinal resection approach; HM: Hand Motion; CD: Counting Fingers.

## Data Availability

The data presented in this study are available on request from the corresponding author. The data (original imaging) are not publicly available due to privacy issues.
