# Correction: Mazzini et al. Anatomical and Functional Outcomes after Endoresection and Adjuvant Ruthenium Brachytherapy for Uveal Melanoma: A Single-Center Experience. *Life* 2023, *13*, 902

**DOI:** 10.3390/life14020162

**Published:** 2024-01-23

**Authors:** Cinzia Mazzini, Giulio Vicini, Laura Di Leo, Daniela Massi, Stanislao Rizzo, Fabrizio Giansanti

**Affiliations:** 1 Unit of Ocular Oncology, Neuromuscular and Sense Organs Department, Careggi University Hospital, 50134 Florence, Italy; cinzia.mazzini@unifi.it (C.M.); laura.dileo92@gmail.com (L.D.L.);; 2 Eye Clinic, Neuromuscular and Sense Organs Department, Careggi University Hospital, 50134 Florence, Italy; 3 Department of Neurosciences, Psychology, Drug Research and Child Health, University of Florence, 50121 Florence, Italy; 4 Section of Pathological Anatomy, Department of Health Sciences, University of Florence, 50139 Florence, Italy; 5 Ophthalmology Unit, Catholic University of the Sacred Heart, Fondazione Policlinico Universitario A. Gemelli, 00168 Rome, Italy; stanislao.rizzo@gmail.com; 6 Consiglio Nazionale delle Ricerche (CNR), 56124 Pisa, Italy

## Text Correction

There was an error in the original publication [1]. The data of a patient included in our series were incorrect (the data of patient #13 shown in Table 1 referred to the situation prior to the initial treatment), and consequently, the reported mean values were incorrect.

The article was edited with the correct data.

The authors state that the scientific conclusions are unaffected. This correction was approved by the Academic Editor. The original publication has also been updated.
